# Shear- vs. nanotopography-guided control of growth of endothelial cells on RGD-nanoparticle-nanowell arrays

**DOI:** 10.1186/1754-1611-7-11

**Published:** 2013-04-22

**Authors:** Katherine E McCracken, Phat L Tran, David J You, Marvin J Slepian, Jeong-Yeol Yoon

**Affiliations:** 1 Department of Agricultural and Biosystems Engineering, The University of Arizona, Tucson, AZ, 85721, USA; 2 Sarver Heart Center and Department of Medicine, College of Medicine, The University of Arizona, Tucson, AZ, 85721, USA; 3 Department of Biomedical Engineering, The University of Arizona, Tucson, AZ, 85721, USA

## Abstract

Endothelialization of therapeutic cardiovascular implants is essential for their intravascular hemocompatibility. We previously described a novel nanowell-RGD-nanoparticle ensemble, which when applied to surfaces led to enhanced endothelialization and retention under static conditions and low flow rates. In the present study we extend our work to determine the interrelated effects of flow rate and the orientation of ensemble-decorated surface arrays on the growth, adhesion and morphology of endothelial cells. Human umbilical vascular endothelial cells (HUVECs) were grown on array surfaces with either 1 μm × 5 μm spacing (“parallel to flow”) and 5 μm × 1 μm spacing (“perpendicular to flow”) and were exposed to a range of shear stress of (0 to 4.7 ± 0.2 dyn·cm^-2^ ), utilizing a pulsatile flow chamber. Under physiological flow (4.7 ± 0.2 dyn·cm^-2^), RGD-nanoparticle-nanowell array patterning significantly enhanced cell adhesion and spreading compared with control surfaces and with static conditions. Furthermore, improved adhesion coincided with higher alignment to surface patterning, intimating the importance of interaction and response to the array surface as a means of resisting flow detachment. Under sub-physiological condition (1.7 ± 0.3 dyn·cm^-2^; corresponding to early angiogenesis), nanowell-nanoparticle patterning did not provide enhanced cell growth and adhesion compared with control surfaces. However, it revealed increased alignment along the direction of flow, rather than the direction of the pattern, thus potentially indicating a threshold for cell guidance and related retention. These results could provide a cue for controlling cell growth and alignment under varying physiological conditions.

## Background

Atherosclerotic coronary and cerebrovascular disease are leading causes of morbidity and mortality around the world [1,2]. Beyond pharmacologic therapy mechanical therapies such as stents, stent grafts, and vascular grafts (artificial vessels) have emerged as major therapeutic options [3-6]. Despite the effectiveness of these approaches limitations remain stemming from bio- and hemocompatibility issues related to adverse device (foreign body)-blood and device-tissue interactions [7-10]. Specifically, the development of thrombosis, inflammation, neointimal thickening and vessel restenosis or reclosure are associated with increased clinical risk [11,12]. To address some of these limitations newer implants have added the feature of local delivery of anti-proliferative drugs, i.e. to modulate excessive smooth muscle cell proliferation associated with neointimal thickening. However, the lack of cell selectivity of current drugs utilized has lead to retardation and delay of implant endothelialization, ultimately essential for implant hemocompatibility [13-15]. A need therefore exists for effective endothelialization strategies, particularly approaches which will lead to enhanced adhesion, growth and retention of cells under physiologic flow conditions [16-18].

Two key factors known to influence endothelial cell adhesion, retention, and orientation on an implant surface are surface topography and overflowing wall shear stress [19,20]. In their native environment, endothelial cells are in contact with the basement membrane, which is formed into a network of submicron- and nanoscale features whose direct interactions with the cells have been linked to improved adhesion, proliferation, and gene regulation [21-23]. Under controlled shear stress, surface topography has been identified as a guiding factor in endothelial cellular orientation and elongation, which is enhanced when patterning is along the constant flow [19,24]. These effects are also dependent upon patterning size, with increased elongation along line features of greater groove-to-ridge ratios [19].

Past studies with flow have indicated that endothelial cells gradually orient and elongate along the direction of fluid flow under uniform shear stress, as evidenced by rapid inclination of cell junctions along the flow, and gradual reorganization of the cytoskeleton, especially with peripheral actin stress fiber formation [25-28]. Such reorientation may benefit the cells by altering the stress profile over the cell surface, by enhancing communication pathways for the conveyance of shearing-induced intracellular molecules, or potentially by improving cell-cell connections and flexibility in the context of a vascular monolayer [20,24,28-32].

While previous work with nanoscale topography has been primarily with bare ridges and grooves, we sought instead to examine the effects of a nanoscale array of etched wells, each electrostatically fitted with an RGD-conjugated nanoparticle to mimic integrin binding sequences of the extracellular matrix on endothelialization. Prior work by our group with these nanowell-RGD nanoparticle ensemble arrays indicated that texture benefitted endothelial cell adhesion and retention more than smooth biocompatible surfaces, even when patterned into minimally cell-adherent PMMA surfaces [33]. With the inherently more-complex surface structure offered by interspersed wells and peptide protrusions, improvement was observed in HUVEC adhesion, spreading, and alignment under shear stress than under similar shear stress across equivalent linear patterning. These morphological characteristics are indicative of healthy cell adaptation to flow conditions [34]. In the present study, we hypothesized that endothelial cell behavior would vary substantially across different directional orientations of an RGD-nanoparticle array under physiological shear stresses associated with flow (in [33], different directional orientations of nanoarray were not investigated, and only very low, sub-physiological wall shear stress conditions were tested). Further, we hypothesized that we might observe a shear “guidance threshold,” i.e. a level at which cell behavior is primarily topography-defined, versus primarily shear-defined. Changes in wall shear stress and the direction of surface patterning were considered and evaluated in order to develop a fuller understanding of the interrelated effects of flow rate and patterning direction on the short-term adhesion, retention, and morphology of HUVECs *in vitro*.

## Results & discussion

### Characterization of surfaces and flow

Five surface structures were fabricated and analyzed including; “parallel to flow” and “perpendicular to flow” configurations of an etched nanowell array affixed with RGD-conjugated nanoparticles; polymethyl methacrylate-coated (PMMA-coated) silicon wafer; boron-doped (p-doped) silicon (+Si); and positively-charged glass (+Glass) as a standard cell culture comparison.

The nanowell arrays, etched by electron beam lithography into PMMA-coated +Si, each featured a 5 μm ×1 μm pattern of 100 nm diameter, 85 nm deep wells that selectively exposed the +Si surface. The parallel and perpendicular orientation of the nanowell array surfaces were defined according to the orientation relative to flow direction, with “parallel” patterning as a 1 μm × 5 μm array, in which flow was directed along the 1 μm row, and with “perpendicular” patterning as a 5 μm × 1 μm array, in which flow was directed along the 5 μm row.

**Figure 1:**
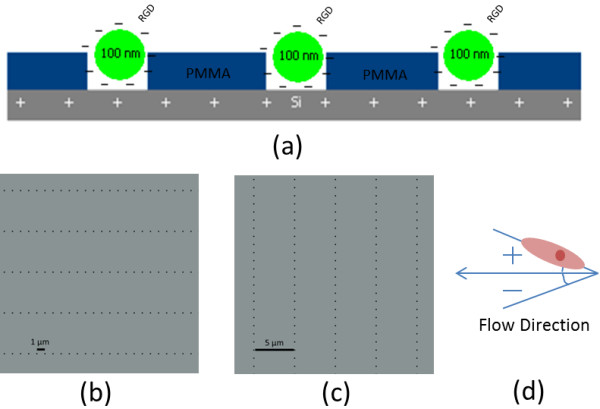
**Nanoarray Surface Patterning.** (**a**) RGD-conjugated nanoparticle array concept. (**b**) Parallel well pattern (1 μm × 5 μm). (**c**) Perpendicular well pattern (5 μm × 1 μm). (**d**) Orientation angle measurement system.

Ensemble arrays of nanowells with electostatically trapped charged-RGD ligand were fabricated by covalently conjugating GRGDSPK (RGD) peptide sequence onto carboxylated, fluorescent polystyrene nanoparticles, followed by selective deposition of these negatively-charged particles into the etched, positively-charged nanowells by electrostatic attraction, according to our previously described method [33] (Figure 1a). In “parallel” orientation, this 5 μm × 1 μm array was established directly along the flow within the flow channel environment (Figure 1b). “Perpendicular” orientation featured this same nanoarray, but etched as a 1 μm × 5 μm pattern, 90˚ to the parallel (Figure 1c).

Before being subjected to flow, HUVECs were incubated under static condition for 24 h, allowing for preliminary adhesion onto the substrate surface. For reference, a set of cells grown over the PMMA and nanowell patterned surfaces were stained and imaged at 24 h. These served as a baseline for visualizing flow-dependent cell retention from 24 h to 48 h. Under the conditions observed, a monolayer had not yet been formed so cell-cell contacts and their effects on cell behavior were not assessed at this time.

**Figure 2:**
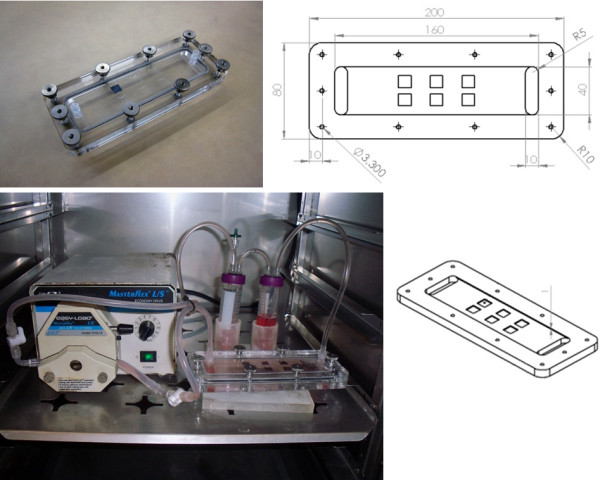
**Bioreactor Design and Characterization.** Thin channel acrylic flow system design with internal cross-section of 4.0 cm × 0.1 cm. Experimental surfaces were placed into individual 1 cm^2^ cut-outs in the lower channel surface so that cell-seeded surfaces were flushed with the wall. Channel was connected in series with a pulsatile pump head and two media reservoirs with gas exchange.

Pulsatile flow conditions were categorized by wall shear stress values at the cell-media interface. Three conditions were studied: static, sub-physiological, and low physiological. Under static condition, the HUVEC-seeded surfaces were retained in a petri-dish environment and static media was exchanged at 24 h. Sub-physiological flow corresponded to an estimated wall shear stress value of 1.7 ± 0.3 dyn∙cm^-2^ (i.e., cycling from 1.4 dyn∙cm^-2^ through 2.0 dyn∙cm^-2^), with variance due to transient changes in flow velocity as a result of the peristaltic pump without a damper. This sub-physiologic flow delivered a media volume of 89 ml∙min^-1^ through a thin-channel flow device of cross-section 4 cm × 0.1 cm (Figure 2). This was connected through a pump and into a two-chamber media reservoir with filtered gas exchange (Figure 2). Low physiologic flow was characterized by wall shear stress of 4.7 ± 0.2 dyn∙cm^-2^ (i.e., cycling from 4.5 dyn∙cm^-2^ through 4.9 dyn∙cm^-2^), with variance also due to transient changes in flow velocity as a result of the peristaltic pumping. This delivered a media flow volume of 239 ml∙min^-1^ within this same flow channel. Each flow condition was sustained for 24 h.

### Surface-dependent endothelial cell retention under flow

Following the initial 24 h seeding, peristaltic flow conditions were introduced from 24 h to 48 h, revealing pronounced differences in HUVEC retention with respect to surface patterning for each level of wall shear stress at the cell-media interface.

**Figure 3:**
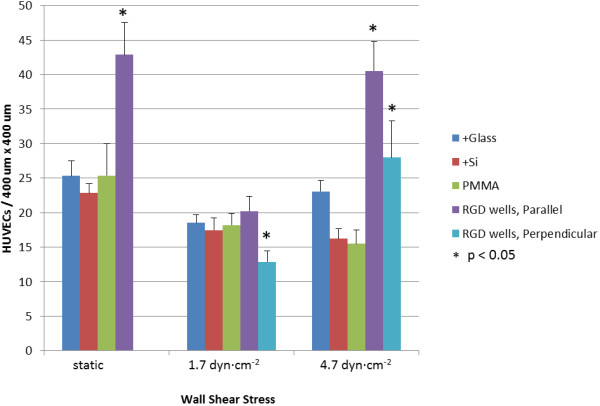
**Cell Retention Relative to Patterning and Shear Stress.** Average cell count per 400 μm × 400 μm unit pattern area, ±1.0σ. Across +Glass, +Si, and PMMA surfaces, cells were counted within a four grid overlay of size equivalent to that of the etched nanowell array. Note: under static condition, patterning is non-directional on account of no flow. * indicates significant difference from the result on PMMA surface (p < 0.05).

**Figure 4:**
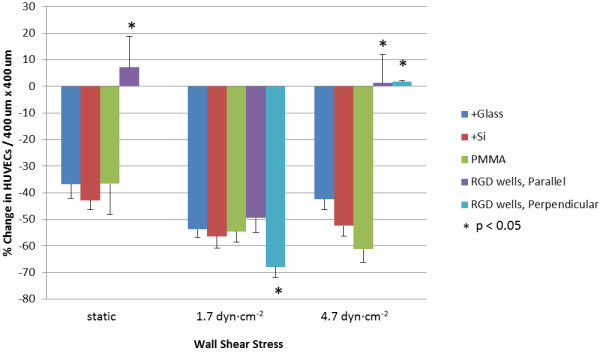
**Cell Retention from 24 h to 48 h Under Flow, Relative to 24 h Static.** Percent change in cell count per 400 μm × 400 μm unit pattern area relative to 24 h static seed, demonstrating surface cell retention. Average cell counts across patterned and PMMA surfaces were subtracted from 28 h static cell counts across all surfaces. Note: under static condition, patterning is non-directional on account of no flow. * indicates significant difference from the result on PMMA surface (p < 0.05).

When static conditions were maintained for the full 48 h, the greatest average cell adhesion per unit pattern area (400 μm × 400 μm) was seen for the RGD-conjugated nanowell surface, with 43 ± 5 HUVECs per pattern – approximately 75% more cells than with the +Glass, +Si, and PMMA control surfaces (Figure 3). When cell counts were re-evaluated relative to the 24 h static incubation baseline, the +Glass, +Si, and PMMA control surfaces saw 36-43% decreases in cell populations, whereas populations over the nanowell surface were the only to experience growth, with a 7% increase at 48 h, which is substantially different from all three control surface (+Glass, +Si and PMMA) with p < 0.05 (static in Figure 4).

When sub-physiological flow was introduced, however, a marked shift was seen in cellular adhesion over all surfaces. Exposed to 1.7 ± 0.3 dyn∙cm^-2^ for 24 h, cell adhesion decreased from their levels under static condition over all surfaces, with similar densities over both hydrophilic +Glass and +Si control surfaces, as well as the hydrophobic PMMA control surface (Figure 3). With the advent of flow, the direction of the RGD-conjugated nanowell array pattern both “parallel” and “perpendicular” to the flow direction became apparent, so cell populations over these two surfaces were independently examined and reported. With 1.7 ± 0.3 dyn∙cm^-2^ wall shear stress, both parallel and perpendicular cell populations decreased from the cell densities seen at 48 h under static conditions. With respect to behavior across the unpatterned control surfaces, however, there was a marked difference, with slightly improved density over parallel patterning, and inferior density over perpendicular patterning. Parallel patterning resulted in 20 ± 2 cells per unit pattern area in total (Figure 3), with an average 49% decrease in population from the 24 h static baseline (Figure 4). Perpendicular patterning resulted in 13 ± 2 cells per unit pattern area (Figure 3), with a 68% decrease under flow when compared to the same 24 h baseline (Figure 4).

Interestingly, with increased, low physiological-level flow conditions and a wall shear stress of 4.7 ± 0.2 dyn∙cm^-2^, patterning greatly enhanced HUVEC adhesion and retention. Whereas cell densities continued to decrease across +Si and PMMA surfaces and only mildly increased over +Glass, cell densities over the parallel and perpendicular patterns each saw a marked increase from those of sub-physiological flow (1.7 ± 0.3 dyn∙cm^-2^), with 41 ± 4 cells and 28 ± 5 cells per unit pattern, respectively (Figure 3). These counts corresponded to an average increase of 1.3% from the static 24 h baseline with the parallel patterning, and a 1.7% increase from this baseline with the perpendicular pattern, both of which are substantially different from all three control surfaces (+Glass, +Si and PMMA), with p < 0.05 (4.7 dyn∙cm^-2^ in Figure 4).

Under static and low-physiological flow conditions, these findings demonstrate that in terms of HUVEC adhesion and retention, RGD-nanoparticle-nanowell arrays enhanced adhesion and retained cells under shear stress, for much the same benefit as ridge-and-groove line patterning [24]. Nanowell patterning also appears to foster cell proliferation, based on the slight increase in cell density above 24 h baseline levels.

Curiously, a sharp decline in HUVEC adhesion was observed for the RGD-nanoparticle composite surface under sub-physiological flow, with poorer adhesion over the perpendicular array surface than over all controls, and with adhesion only mildly better over the parallel array (Figure 4). Intuitively, surface cell loss would seem correlated with higher wall shear stress due to increased force on the cells. With higher low-physiological flow, however, cell retention was enhanced. This behavior may indicate the interplay of cell adhesion, cell orientation and cell-surface interactions. Principally, with higher physiologically-relevant shear stress (4.7 ± 0.2 dyn∙cm^-2^), cell flattening with relation to the underlying pattern orientation was observed, as be discussed in the following sections. Such characteristics were not observed to equal extent under lower sub-physiological shear stress, or over non-patterned control surfaces under the same low physiological shear stress. This suggests that patterning under physiologically-relevant shear stress facilitates cell stress-relieving mechanisms, as have been similarly observed and described over linear ridge-and-groove surface patterning [19].

### Cellular orientation relative to flow and patterning direction

HUVECs adhered to each surface at 48 h were also measured for their orientation relative to the flow direction, as taken by the offset angle between the direction of flow and the long axis of their best-fit ellipse (Figure 1d).

**Figure 5:**
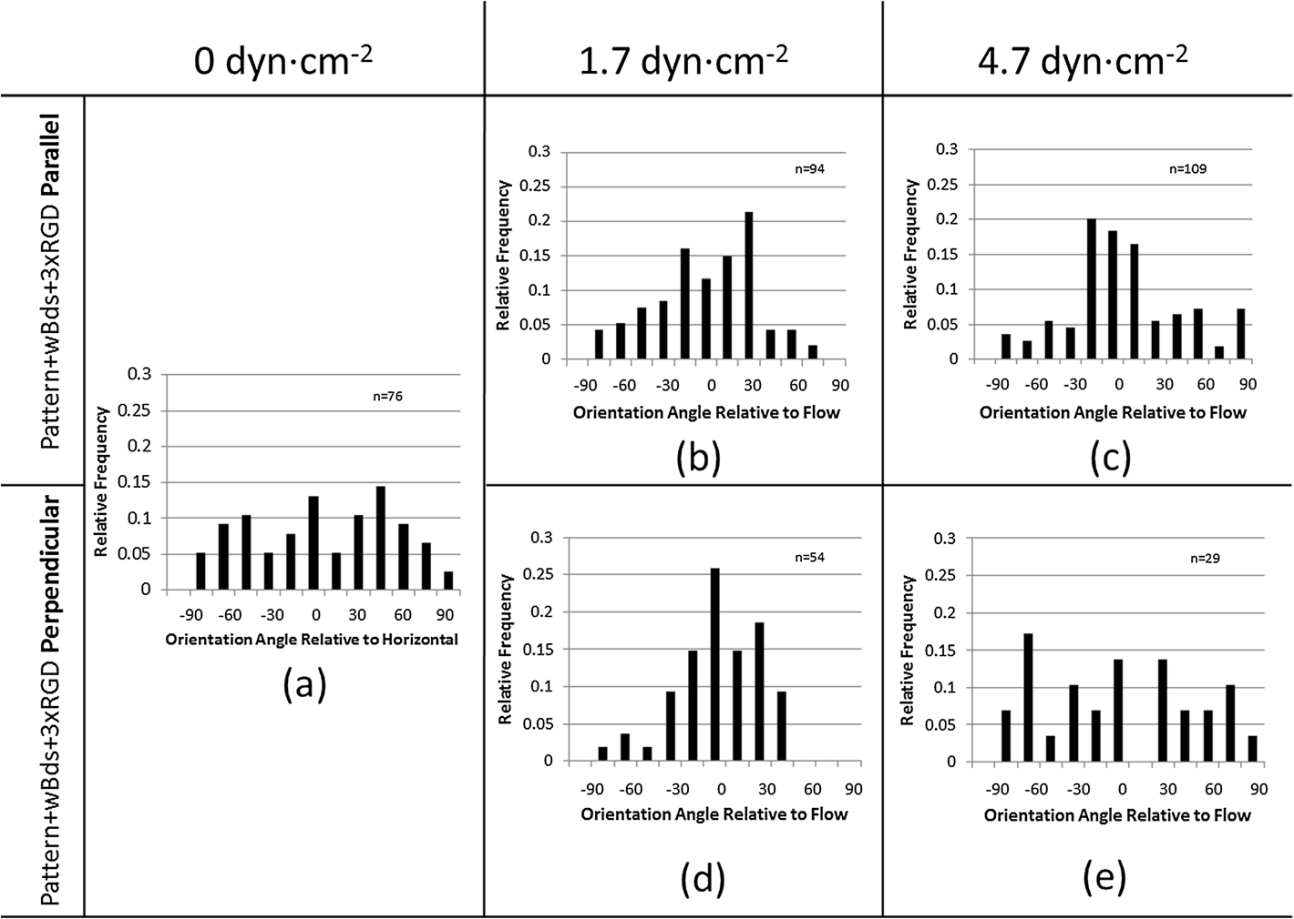
**Cell Orientation Relative to Patterning and Flow Direction.** Orientation of HUVECs across directional nanowell surface topography under sequential flow conditions. Angle measurements were taken as the long axis of each ellipse-fitted HUVEC relative to the flow direction, or to the horizontal in the case of 48 h static condition.

Across the parallel and perpendicular patterns, the distribution of the cells’ orientation angles carried a trend that aligned with cell retention data, suggesting a linkage between these factors in relation to flow resistance. Namely, cell orientation along the surface patterning appeared to correspond more with improved cell retention, while orientation primarily along the flow axis corresponded with relatively lesser retention. Under sub-physiological flow (1.7 ± 0.3 dyn∙cm^-2^), cell retention over the RGD-nanoparticle arrays was poorest and resembled that over the PMMA, +Si, and +Glass surfaces. At the same time, cells aligned predominantly along the flow axis across both patterns – surprisingly, more so on perpendicular patterning than on parallel patterning (Figure 5b,d). This may indicate greater responsiveness to flow, rather than patterning, under these flow conditions. When HUVECs were exposed to low physiological flow levels (4.7 ± 0.2 dyn∙cm^-2^), however, the alignment trend shifted. While cells over parallel patterning saw improved alignment ±15° from the flow axis, cells over perpendicular patterning saw a drastic shift in behavior, with alignment more evenly distributed between the flow and the patterning directions (Figure 5c,e). Under 48 h static condition, cell alignment appeared evenly distributed across both patterns (Figure 5a).

The connection between cell orientation and flow may display one defining benefit of this nanoparticle-nanowell array. Under sub-physiological flow, cell orientation was responsive to flow direction across both patterns; under low physiological flow, cells became more responsive to patterning direction. This may indicate that increasing shear stress encourages cell-surface interactions with the topography or RGD peptide; i.e. cells align themselves along the flow direction in order to decrease the effective force felt by the cells [19]. While in the case of perpendicular patterning, alignment against the flow may not be ideal for this very reason, this behavior suggests a sort of adhesion and guidance threshold between the 1.7 ± 0.3 dyn∙cm^-2^ of sub-physiological flow, when flow directional effects dominate, and the 4.7 ± 0.2 dyn∙cm^-2^ of low physiological flow, when surface patterning effects dominate.

As with conventional ridge-and-groove nano-/micro-scale patterning, directional patterning of the RGD-nanoparticle array modulates cell orientation behavior with respect to flow direction. Under normal and high physiological shear stresses of 12–40 dyn∙cm^-2^, linear ridges and grooves have been seen to enhance elongation and to guide cell orientation along the patterning direction to within 10–20° of patterns parallel to flow, and to within 20-50° of patterns perpendicular to flow, depending on feature ratios [19,24]. Similarly, the RGD-nanoparticle array encouraged cell orientation, with greater correspondence to patterning direction under low physiological flow (Figure 5). This directional control, coupled with improved cell adhesion over an otherwise unfavorable PMMA surface, suggests that the principle of an RGD-nanoparticle array may be employed to enhance surfaces for biocompatibility. Guidance through RGD-nanoparticle-filled nanowells, however, may help to maintain even surface coverage because there are no grooves to partially confine the endothelial cells. Furthermore, surfaces may be more precisely modulated through the spatial controllability of nanoparticles. While the benefits of linear ridge-and-groove patterning and the RGD-nanoparticle array appear to be similar, RGD-nanoparticles provide the additional benefit of post-fabrication control over the spatial characteristics of the surface. Previous work with this array has indicated that nanowells unbound to RGD-nanoparticles do not bear the same cell adhesive benefits of the full RGD-nanoparticle array [33]. Therefore, through control of RGD-nanoparticle deposition, an further control over cell adhesion may be added.

### HUVEC surface spreading and coverage

Cell morphology was evaluated and visible spreading of cells was determined. This was important as greater cell counts though with low surface interaction – as reflected in HUVEC rounding, rather than spreading – by no means suggests optimal cell fitness across a surface [34]. In order to demonstrate the effectiveness of patterning in promoting optimum cell morphology and retention, focus was given to differences in an unpatterned PMMA surface and a parallel patterned RGD nanoparticle-nanowell array.

**Figure 6:**
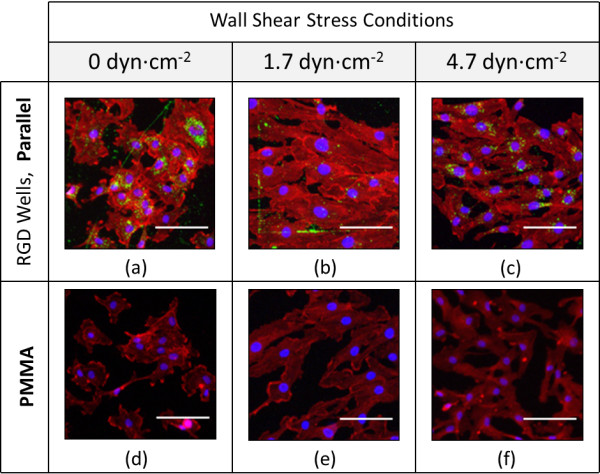
**HUVEC Morphology Relative to RGD Well Patterning and Flow Conditions.** Representative morphology of HUVECs across the Parallel RGD Well pattern and unpatterned PMMA surfaces. HUVECs between passages 3 and 6 were seeded statically for 24 h before being subjected to the listed flow conditions from 24 h to 48 h. Scale represents 20 μm.

Under static condition, cells over the PMMA surface experienced lower attachment, and slightly lower surface spreading when compared with the parallel RGD nanoparticle-nanowell array (Figure 6). The average coverage area of cells over the PMMA surface decreased from 65% under static condition to 29% under 4.7 ± 0.2 dyn∙cm^-2^. In contrast, parallel surface patterning maintained cell coverage under physiologically-relevant flow: 56% under static condition and 56% under 4.7 ± 0.2 dyn∙cm^-2^. The latter value, 56% is largely identical to the average coverage areas of cells over the hydrophilic +Glass and +Si control surfaces under 4.7 ± 0.2 dyn∙cm^-2^, 66% and 55%, respectively. (Due to interactions between the net positive charge of +Glass and +Si and the net negative charge of the cell membrane, cell coverage was expected to be maximum.) This result indicates that the HUVECs can spread on the parallel surface patterning (mostly hydrophobic PMMA) at an extent comparable to that on cell-favourable +Glass or +Si, under low physiologically-relevant flow levels. This indicates the benefit of this RGD nanoparticle-nanowell array for augmenting cellular adhesion under physiologically-relevant flow, with optimum interaction to the surfaces that traditionally incite poor contact.

Advantages for cell coverage on RGD-nanoparticle array surfaces are thus seen primarily at low physiologically-relevant flow levels. Under such conditions, deliberate patterning may be used to transform a specific section of a given biologically unfavorable surface – here PMMA – into an exceptionally favorable center for adhesion and cell-surface interaction, improved even above a standard hydrophilic cell culture surface.

## Conclusions

An array of ensemble nanostructures consisting of RGD-conjugated nanoparticle-nanowells offers complex nanoscale surface texture features for enhancing HUVEC adhesion under both static and low physiological shear stress conditions, comparable to those of resistance vasculature, and potentially early angiogenesis. Additionally, while it offers nanoscale topography, it possesses characteristics that significantly differ from those of linear ridge-and-groove sub-micron and nanoscale patterning, a frequently-studied fabricated surface structure. Across the nanoparticle-nanowell pattern, cells are retained above and across the upper level of the pattern surface, perhaps due to the lack of exposed subsurface space in the nanoparticle-filled nanowells. Even still, endothelial cells adhere and are directionally-guided as experienced in similar ridge-and-groove linear patterning [19,24]. At the same time, for static and low physiological flow conditions, this nanoarray offers enhanced adhesion and retention across an otherwise non-biologically-favorable PMMA surface. HUVECs were aligned along the flow in sub-physiological wall shear stress (1.7 dyn·cm^-2^) while under low physiological wall shear stress (4.7 dyn·cm^-2^) they were aligned along the pattern, potentially to decrease the effective force felt by the cells.

These results provide a cue for topographic features that may control cell growth and alignment under varying physiological conditions; i.e. with flow shear controling cell guidance and retention under conditions associated with sub-physiological shear stress, e.g. angiogenesis, while nanotopography dominates under higher levels of physiological shear stress.

These findings speak of the possibilities for employing a nanowell-nanoparticle pattern as an alternative surface for improving endothelial cell adhesion, retention, and selective guidance when static and physiological-level flow conditions are considered. This would be particularly applicable in a conditioning process for surfaces to be introduced into the physiological environment. The appearance of improved cell retention on the parallel patterned surface under 4.7 dyn∙cm^-2^ wall shear stress may even speak of a great potential for directionally-minded surface patterning in enhancing the “appeal” of typical vascular implantable surfaces that may be impeding the natural post-implant healing process or potentially disrupting the system further, eliciting increased clotting, as may be the situation with some bare metal and drug-eluting stents.

## Methods

### Surface patterning and preparation

The nanoscale patterned surface was first prepared by spin-coating a 2:3 mixture of PMMA:chlorobenzene thinner (Microchem, Newton, MA, USA) onto an acetone-cleaned 1 cm × 1 cm p-doped silicon wafer (boron-doped 111 Si, 450-648-μm thick and 4–75 Ω·cm^-1^, Exsil, Inc., Prescott, AZ, USA). Spin-coating was completed in a two-step process at 400 rpm for 8 s, and 4000 rpm for 25 s, for a measured thickness of 80 nm using a KLA-Tencor Alpha-Step 200 profilometer (Milpitas, CA, USA) and Veeco Dimension 3100 atomic force microscope (AFM; Bruker AXS, Santa Barbara, CA, USA). A series of ten 400 μm × 400 μm array patterns of 100-nm diameter wells spaced at 5 μm × 1 μm were then patterned into this PMMA surface via electron beam lithography, using an FEI Inspec scanning electron microscope (SEM; Hillsboro, OR, USA) equipped with nanometer pattern generating system (NPGS; JC Nabity, Bozeman, MT, USA). This pattern was characterized and validated through SEM and AFM imaging.

Just prior to seeding cells onto the array, the pattern was developed in a three-step process of immersion in 1:3 mixture of methyl isobutyl ketone (MIBK; Microchem Corp., Newton, MA, USA) and isopropyl alcohol (IPA; Honeywell, Morristown NJ, USA) for 1 min, IPA for 30 s, and a deionized water rinse before drying the chips under nitrogen (N_2_) gas. Onto this developed pattern, 100-nm polystyrene nanoparticles (carboxylated and fluorescent) conjugated with RGD were distributed onto the wells.

Conjugation of RGD to the nanoparticles was carried out according to a revised general protein coupling workflow protocol (Bio-Rad, Hercules, CA, USA). In this, 1 ml of 0.02% (w/v) 0.1 μm FluoSpheres fluorescent polystyrene nanoparticles (Invitrogen, Eugene, OR, USA) were conjugated with 42.7 μl of a 1 mg·ml^-1^ solution of the GRGDSPK (RGD) peptide sequence (Anaspec, Inc. Fremont, CA, USA) according to the protocol delineated by Tran et al. [33].

RGD-conjugated nanoparticles were distributed across the array through micropipetting, with three passes of a 1 μl droplet of the RGD-nanoparticle suspension over each developed nanowell array pattern. This was followed by a double rinse of PBS. Entrapment of the negatively charged RGD-conjugated nanoparticles through electrostatic attraction to the positively charged wells was confirmed using an FEI Inspec SEM.

### Preliminary cell culturing

Human umbilical vascular endothelial cells (BD Biosciences, San Jose, CA, USA) were grown in M199 medium (Lonza Biowhittaker, Walkersville, MD, USA), supplemented with 15% (v/v) fetal calf serum (Lonza Biowhittaker); 1% (v/v) each of 200 mM L-glutamine (Lonza Biowhittaker), 1 M HEPES (Lonza Biowhittaker), and penicillin:streptomycin (10,000 U∙ml^-1^ : 10,000 μg∙ml^-1^, Lonza Biowhittaker); 25 mg of endothelial cell growth supplement (ECGS; Biomedical Technologies, Inc., Stoughton, MA, USA) and 26.4 mg heparin sodium, for 500 ml total mixed medium. HUVECs were cultured within a bioreactor set at 37°C, 5% CO_2_ until they reached approximately 80% confluency within a gelatin-coated culture flask (dextrose gelatin veronal; Biowhittaker, Lonza, Basel, Switzerland) culture flask, and were used between passages 2–6.

### Cell culturing under flow conditions

At 80% confluency, cells were passaged using 2 ml of a 1:1 mixture of trypsin and Hank’s Balances Salt Solution (HBSS, without Ca^2+^ or Mg^2+^; Lonza Biowhittaker). Cells were resuspended at 350,000 cells∙ml^-1^, of which 0.2 ml was seeded onto each patterned chip surface for 10 min. These chips were then fully immersed in the M199 media and left to incubate statically for 24 h in the bioreactor. Following this incubation period, the chips were placed into the designated flow channel which was fed from a system of two 50 ml reservoirs, the first with filtered gas exchange, filled with 20 ml and 30 ml of the mixed M199 media. Media was drawn through the channel and silicone connective tubing (Masterflex® platinum-cured silicone tubing, L/S®; Cole-Parmer, Vernon Hills, IL, USA) by peristaltic pumping action (Cole-Palmer). Cells were then incubated under flow for 24 h. For the 48 h static controls, the static seeded chips were indirectly rinsed once with PBS, and their media was replenished.

### Channel design and flow conditions

Flow was applied to the seeded chip surfaces within a two-piece acrylic thin-channel device of dimensions 16 cm × 4 cm × 0.1 cm (Figure 2). Square grooves of dimensions 1 cm × 1 cm × 0.5 cm were machined into the lower interior face, allowing the chips to be positioned in such a way that the seeded surface would be flush with the wall.

The pump was set to produce average flow rates ranging between 89–239 ml∙min^-1^ for 24 h within the bioreactor. The direction of flow was marked by a small scratch along each chip, away from the pattern, which was later used for reference in analysis.Wall shear stress values for all flow conditions were estimated based on the relationship τ = (6μQ) / (H^2^W) with μ referring to the dynamic viscosity of the M199 mixed media, μ = 0.782 mPa∙s^-1^[6], Q referring to the volumetric rate of media flow through the channel, and H and W being the height and width dimensions of the flow channel’s cross section.

The pulsatile action of the pump (without a damper) caused the flow rate and subsequently the wall shear stress to suffer a time dependency. This time dependency was determined through measuring pressure changes in the channel. These pressure changes were correlated to velocity change through Bernoulli’s principle, then to the flow rate variance and finally to the wall shear stress using the above equation.

### Cell adhesion and orientation

At 24 h, the chips were removed from the channel and cells were stained for immunofluorescence using an actin cytoskeleton and focal adhesion staining kit (Millipore, Billerica, MA, USA) according to the standard Millipore protocol. In this, HUVECs were fixed using 4% para-formaldehyde for 15 minutes, and then washed in 0.05% Tween 20 and perforated by applying a 0.05% Triton X solution for 5 minutes. Following a second washing step, non-specific antigen binding was blocked using a 0.5% bovine serum albumin (BSA; Sigma-Aldrich, St. Louis, MO, USA) solution for 15 minutes. Vinculin was then fluorescently labeled using fluorescein isothiocyanate conjugatedmouse anti-immunoglobulin G (mIgG-FITC) for 1 hour, followed by a washing step. F-actin was stained next using tetramethyl rhodamine isothicyanate (TRITC) conjugated Phalloidin. After 2 final washing stages, cells were mounted for imaging in a vector shield containing DAPI (Vector Laboratories, Inc., Burlingame, CA, USA). Cells were then imaged at 10x using epifluorescence and confocal microscopy.

Using ImageJ software (National Institutes of Health, USA), the angles of orientation were measured relative to the flow direction according the system detailed in Figure 1d and the cell numbers were counted per unit patterning area: 400 μm × 400 μm. Over patterned surfaces, this unit area was defined by the evident boundary between the fluorescent green nanoparticle array and the surrounding unpatterned PMMA. Over the +Glass, +Si, and PMMA surfaces, this unit area was defined by a 400 μm × 400 μm grid overlay applied to each image. In each image, only full arrays were considered, and only cells with a visible nucleus and with no visible sign of necrosis or apoptosis that were fixed over the array after 24 h were counted. For analysis of orientation, angle was measured for the long axis of the cell body relative to the flow direction, as determined to be lengthwise along the widest segment of the cell body containing the nucleus, discounting apparent filopodia.

### Percent coverage of pattern

Over these same defined array and grid areas, gray scale and red scale histograms were collected and the average pixel intensity was taken for each measurement within the ImageJ software (National Institutes of Health, USA). Similarly, gray scale and red scale histograms were taken for null regions and regions of full cell coverage of each image, and the average pixel intensities were also measured. Data for the array and grid areas were averaged within each image, and were then compared relative to the controls for their respective image. Percent coverage of cells was evaluated for a given array (or grid).

## Competing interests

The authors declare that they have no competing interests.

## Authors' contributions

KEM performed surface patterning and preparation, SEM/AFM imaging, cell culture with bioreactor, fluorescent/confocal microscopic analyses, and ImageJ analyses, with assistance from PLT. DJY designed and fabricated the biochamber. JYY conceived the original idea in discussion with KEM, PLT and MJS. All experimental results were analyzed by KEM, PLT, MJS and JYY. KEM wrote the first draft of the manuscript that was revised by JYY and MJS. All authors read and approved the final manuscript.
